# Mycotic aneurysm caused by *Edwardsiella tarda* successfully treated with stenting and suppressive antibiotic therapy: a case report and systematic review

**DOI:** 10.1186/s12941-018-0273-x

**Published:** 2018-05-10

**Authors:** Kei Furui Ebisawa, Sho Nishimura, Shungo Yamamoto, Goh Ohji, Kentaro Iwata

**Affiliations:** 1 Division of Infectious Disease, Kobe University Hospital, 7-5-2 Kusunoki-cho, Chuo-ku, Kobe, Hyogo 650-0017 Japan; 2 Integrated Clinical Education Center, Kyoto University Hospital, 54 Kawaharacho, Shogoin, Sakyo-ku, Kyoto, 606-8507 Japan

**Keywords:** *Edwardsiella tarda*, Cirrhosis, Mycotic aneurysm, Stent graft, Suppressive antibiotic therapy

## Abstract

**Background:**

Mycotic aneurysm is an uncommon disease which could be fatal without appropriate treatment. Although standard therapy for mycotic aneurysms consists of resection of the infected aorta and in situ graft replacement, some treat with endovascular stent-grafting because patients may not tolerate graft replacement due to underlying diseases. There are 6 more reported cases of mycotic aneurysm caused by *Edwardsiella tarda*. With the exception of our case, all underwent resection and debridement of the infected aorta or vascular prosthesis. Herein we report the first case ever of mycotic aneurysm caused by *E. tarda*, successfully treated with stenting and suppressive antibiotic therapy without resection of the infected aorta.

**Case presentation:**

A 65-year-old Japanese woman with cirrhosis and hepatocellular carcinoma complained of fatigue. Her work up revealed a ruptured aneurysm of the descending aorta. She went through endovascular stent-graft placement. *Edwardsiella tarda* grew from blood cultures, which led to the diagnosis of mycotic aneurysm. *Edwardsiella tarda* is a Gram negative bacillus which rarely causes infections in humans. In the case of bacteremia, its mortality is reported to be very high and all reported cases with mycotic aneurysm caused by *E. tarda* ended up with resection of the infected aorta.

**Conclusion:**

Our case shows that in the case of mycotic aneurysm caused by *E. tarda*, endovascular stent-graft placement could be an alternative to in situ graft replacement.

**Electronic supplementary material:**

The online version of this article (10.1186/s12941-018-0273-x) contains supplementary material, which is available to authorized users.

## Background

*Edwardsiella tarda* is a Gram negative bacillus which is usually found in fresh water and the stools of reptilians such as fish, lizards, snakes, alligators, and so on [1]. Human infections by *E. tarda* are rare and many of them are associated with gastrointestinal diseases [1]. In this study, we aimed to systematically review the characteristics of mycotic aneurysm caused by *E. tarda*, and report a case of mycotic aneurysm caused by *E. tarda* successfully treated with stenting and suppressive antibiotic therapy.

## Materials and methods

We searched PubMed, Google scholar (GS), and Ichushi-Web (Japan Medical Abstracts Society: JAMAS) from inception to March 14, 2018. Each search query included the terms *Edwardsiella tarda*, and aneurysm, or vascular prosthesis, or stent graft. In Ichushi-Web and GS, these terms in Japanese characters were also used for search (Supplementary appendix). Articles were assessed for inclusion independently by two reviewers (S. Y. and K. E.).

## Results

**Fig. 1:**
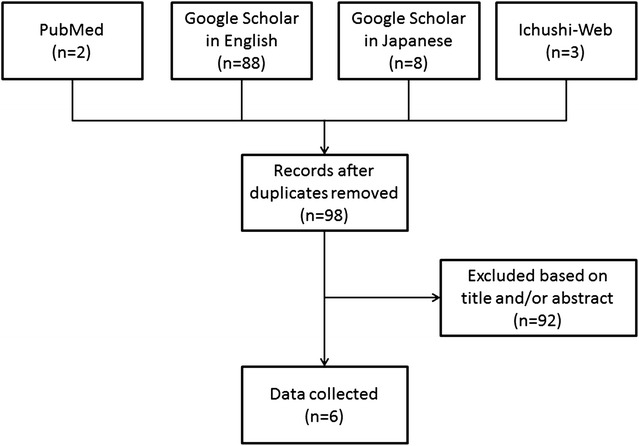
Flowchart of literature search

101 articles were identified (PubMed 2, GS 88 in English, 8 in Japanese, Ichushi-Web 3) and were assessed for inclusion independently by two reviewers (S. Y. and K. E.). 95 articles were excluded based on title and/or abstract or due to duplication. There are 6 more reported cases of mycotic aneurysm caused by *E. tarda* (Fig. 1).

## Case presentation

**Fig. 2:**
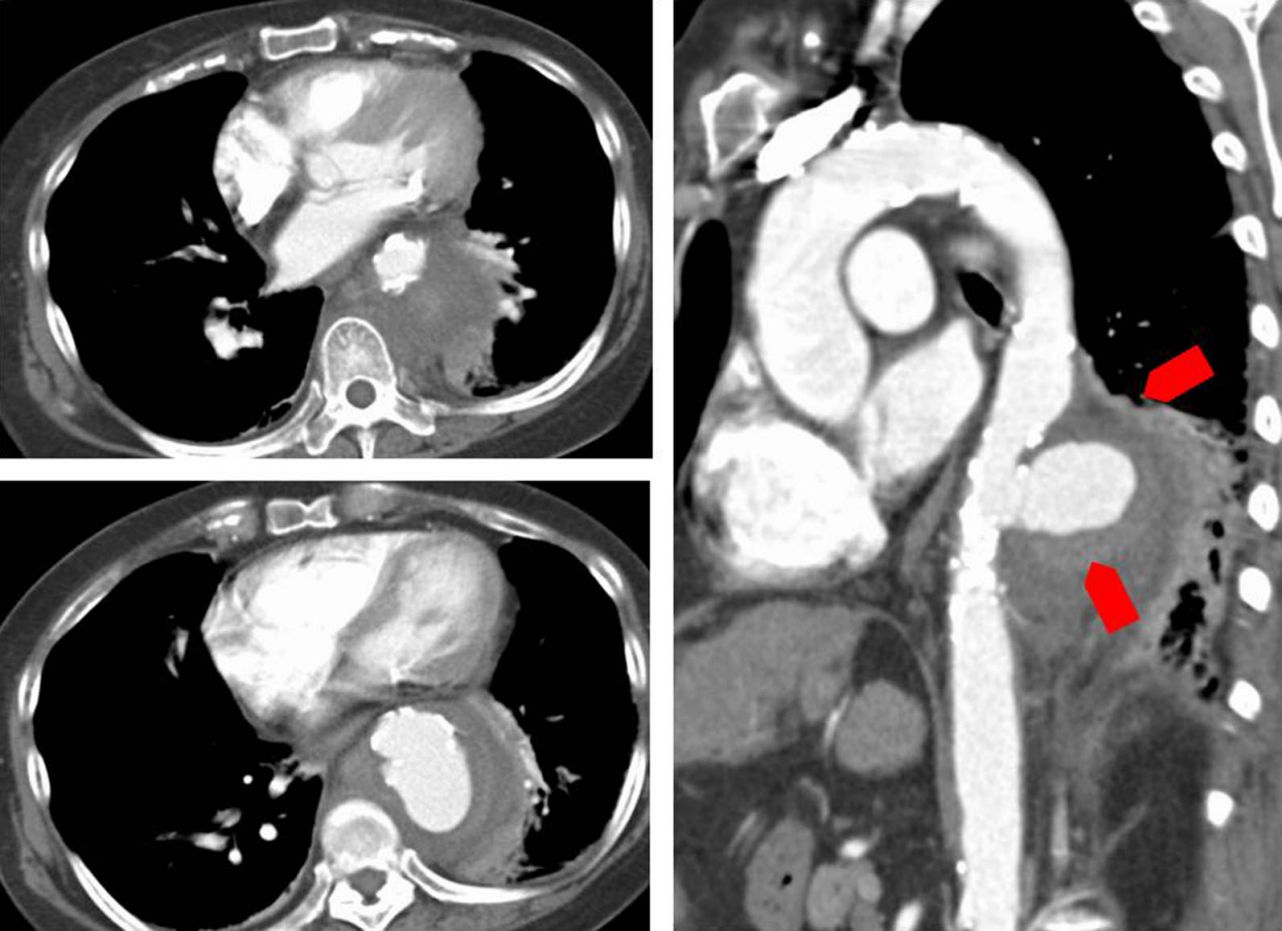
Contrast enhanced CT scan of the descending aorta on admission. There is a ruptured aortic aneurysm of the descending aorta

A sixty five-year-old Japanese woman with cirrhosis, hepatocellular carcinoma, chronic heart failure, pulmonary hypertension and diabetes mellitus presented with fatigue and diarrhea. Three weeks prior to admission she had developed diarrhea and general fatigue. She visited another hospital and computed tomography (CT) revealed a ruptured aneurysm of the descending aorta. For further surgical work up and treatment, she was transferred to our hospital. Physical examination was unremarkable except for a low grade fever of 37.9 °C. Laboratory examination detected an elevated white blood cell count and C-reactive protein. Her Child–Pugh score was 9 points out of 15 (Child–Pugh: B). Chest CT revealed a ruptured aneurysm of the descending aorta of 58 × 47 mm in size (Fig. 2). Based on the imaging study and presence of inflammation, we made a diagnosis of mycotic aneurysm.

**Fig. 3:**
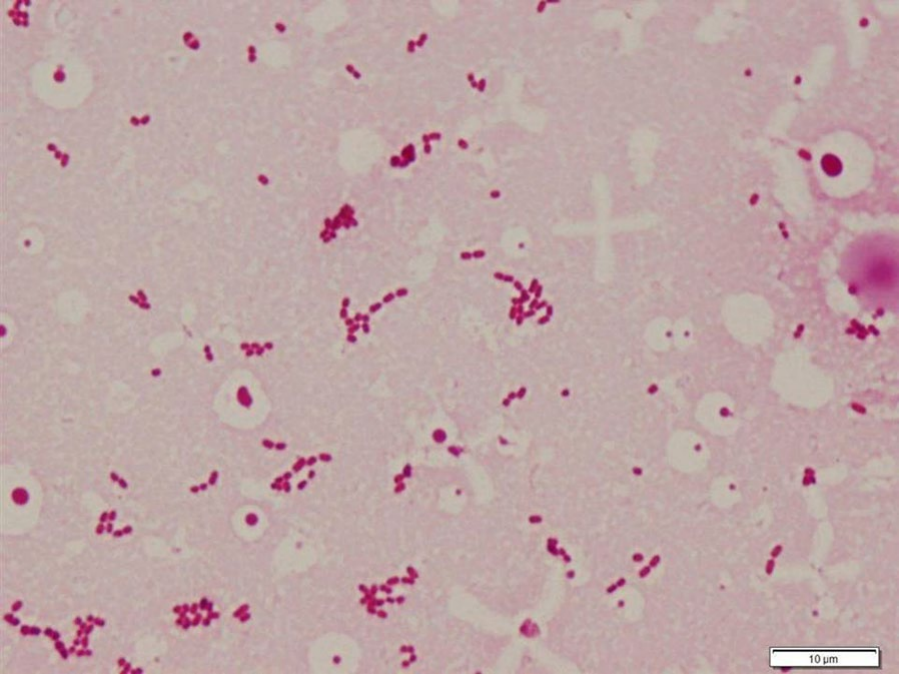
Gram stain of blood culture. Small gram negative rods were seen

**Fig. 4:**
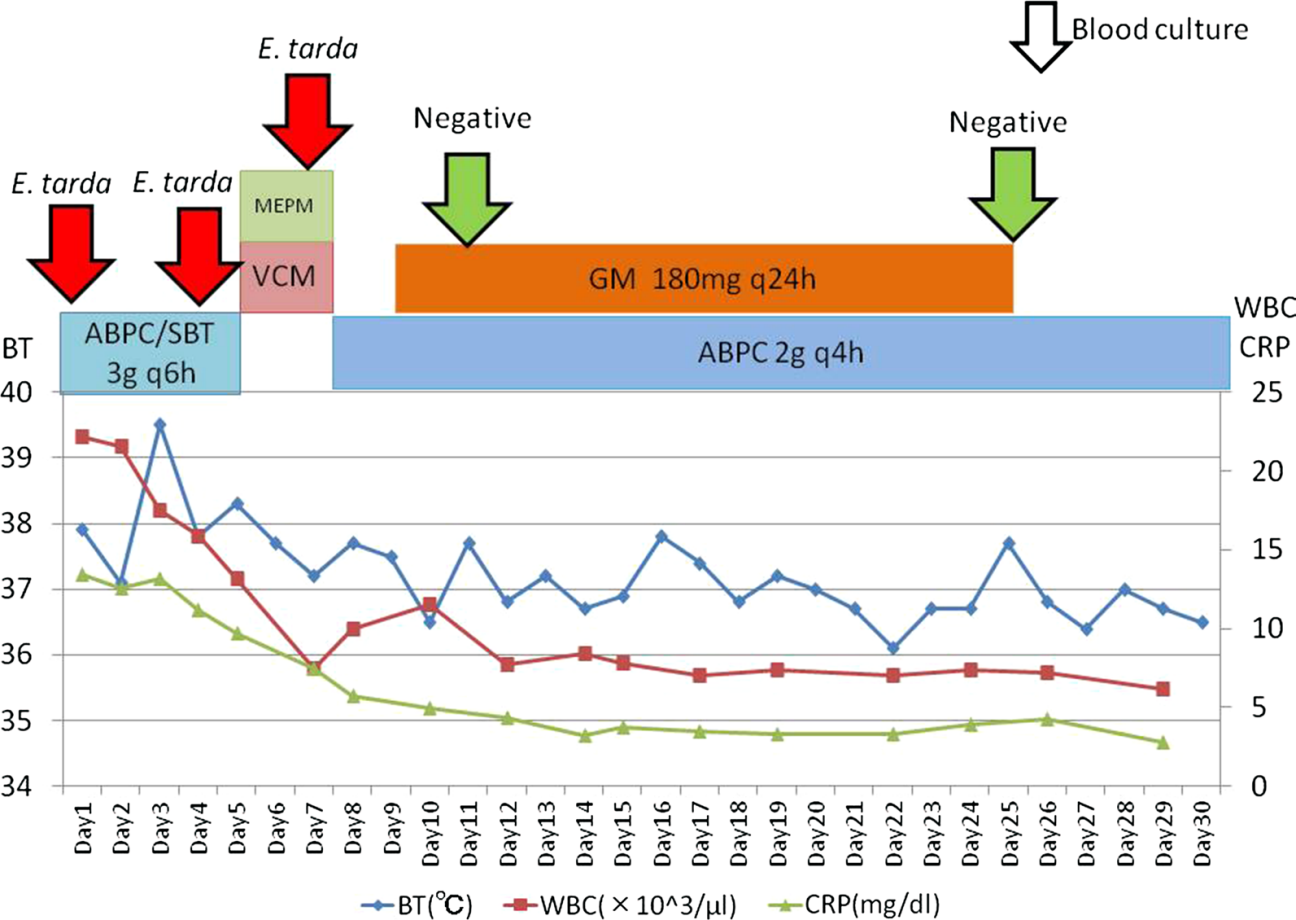
Clinical course until day 30. *ABPC* ampicilline, *SBT* sulbactam, *MEPM* meropenem, *VCM* vancomycin, *GM* gentamicin. The patient’s hypotension forced us to switch to meropenem/vancomycin temporarily

Although the principal treatment for mycotic aneurysm is surgical resection and debridement with antibiotics, thoracic endovascular aortic repair (TEVAR) was performed, due to her high risk of perioperative death. We treated her with ampicillin/sulbactam 3 g every 6 h. 3 days after admission, *E. tarda* grew from her blood cultures (Fig. 3). *E. tarda* was identified using MicroScan WalkAway and Matrix Assisted Laser Desorption/Ionization Time of Flight Mass Spectrometry (MALDI TOFMS). Despite treatment with antibiotics, the fever persisted and the organism continued to grow from blood cultures. We switched antibiotics to vancomycin and meropenem because of clinical deterioration and persistent bacteremia. *E. tarda* turned out to be susceptible to amoxicillin, cephalosporin, aminoglycosides, and quinolones. On day 8, we changed antibiotics to ampicillin 2 g every 4 h as de-escalation. On day 9, we added gentamicin because the blood culture taken on day 7 was again positive, which finally led to negative blood cultures on day 11. We conducted two weeks of dual therapy with ampicillin and gentamicin, followed by 4 weeks of monotherapy with ampicillin (Fig. 4). After a total of 6 weeks of intravenous antibiotic therapy, we switched to oral amoxicillin as suppressive therapy. She was transferred to another hospital 58 days after TEVAR.

Four months later, the patient inadvertently discontinued her antibiotic therapy for a week, which led to recurrence of fever again. She was admitted to another hospital, and *E. tarda* was confirmed again from blood cultures. She was treated with intravenous ampicillin for another 6 weeks and restarted on suppressive therapy with oral amoxicillin. The antibiotic susceptibility of *E. tarda* did not change from the first strain. Two months later, she visited our hospital with hemoptysis. A broncho-aortic fistula was confirmed and we performed another stent graft treatment. Blood cultures, which were taken at this time, were sterile. We started treatment with ampicillin/sulbactam 3 g every 6 h, and after 6 weeks of intravenous therapy, changed to oral amoxcillin/clavulanate. 3 years after this episode as of this writing, there has been no recurrence of symptoms after the second stent insertion.

## Discussion and conclusions

We presented a rare case of mycotic aneurysm caused by *E. tarda*. All previously reported cases required resection and debridement of the infected area. Our case was successfully treated with stent graft insertion and prolonged antibiotic therapy without resection and debridement. Endovascular stent grafting might be an alternative therapy in patients who were not candidates for surgical replacement due to underlying diseases.

*E. tarda* is a member of the Enterobacteriacae, which is commonly isolated from reptilians, fish, amphibians, and also from fresh water [2]. It was first described by Sakazaki et al. in 1962 [3], at the meeting of the Japan Bacteriological Society, as a new group of the family Enterobacteriacae, the “Asakua group” and in 1965 a summary of the presentation was published in English [4]. In 1964, King et al. described the same species as Bartholomew group [5]. In 1965, Ewing et al. summed up those groups to *Edwardsiella tarda* in honor of the American bacteriologist P. R. Edwards [6]. *E. tarda* rarely causes infection in humans, and the first case series was published in 1969 [7]. After that, some cases of *E. tarda* infections were published, many of which were gastrointestinal diseases [1]. It also causes wound infections, abscesses, and bacteremia, sometimes in association with marine exposure [8].

There are 6 more reported cases of mycotic aneurysm caused by *E. tarda* [9–14] (Table 1). Three of these cases were infections of vascular prosthesis [10, 13, 14]. With the exception of our case, all underwent resection and debridement of the infected aorta or vascular prosthesis. Including the present case, the mean age of the cases was 66.6 years. Three cases had back pain at onset [9–11], and two cases had another site of inflammation such as cellulitis [12] or prostatitis [13] although our case complained only of general fatigue. Blood cultures were positive for two cases and tissue cultures were positive for four cases. We couldn’t get any information about blood culture or tissue culture for one case [12]. In this case, *E. tarda* was cultured from pus of his left leg, and he was diagnosed as mycotic aneurysm from the clinical course and pathological findings. Antibiotic choices for treatment and duration of therapy varied. One patient died 7 months after surgery because of homograft rupture [11]. All other cases, including the present case, had a good prognosis.

**Table 1:** Cases of mycotic aneurysm caused by *E. tarda*

Age/sex	Symptoms	Site of infection	Treatment strategy	Culture	Antibiotics	Duration of antibiotics	Prognosis	References
65 y.o./woman	General fatigue	Descending aorta	Thoracic endovascular stent-graft (TEVAR)	Blood: positive Tissue: not taken	A/S ⇒ MEPM + VCM ⇒ ABPC + GM ⇒ AMPC ⇒ A/C	Suppression	Survived	Present case
79 y.o./Man	Back pain	Aortal arch	Vascular prosthesis implantation	Blood: negative Tissue: positive	CTRX + VCM ⇒ ST ⇒ MINO	Suppression	Survived	[9]
69 y.o./man	Back pain Bloody stools	Vascular prosthesis of abdominal aorta	Vascular prosthesis reimplantation	Blood: negative Tissue: positive	VCM + GM + MTNZ ⇒ PCG + GM + MTNZ ⇒ IPM/CS ⇒ A/C	4 months	Survived	[10]
67y.o./man	Back pain	Abdominal aorta	In situ cryopreserved homograft replacement	Blood: positive Tissue: positive	IMP/CS + FOM ⇒ CPFX	4 months	Dead	[11]
65 y.o./man	Cellulitis	Ascending aorta, Abdominal aorta	Vascular prosthesis implantation	Blood: ND Tissue: ND	ND	ND	Survived	[12]
60 y.o./man	Dysuria	Vascular prosthesis of ascending aorta	Vascular prosthesis reimplantation	Blood: positive Tissue: negative	CTRX ⇒ LVFX	ND	Survived	[13]
61y.o./man	ND	Vascular prosthesis of ascending aorta	Vascular prosthesis reimplantation	Blood: ND Tissue: positive	ND	ND	Survived	[14]

*A/S* ampicillin/sulbactum, *MEPM* meropenem, *VCM* vancomycin, *ABPC* ampicillin, *A/C* amoxicillin/clavulanate, *GM* gentamicin, *AMPC* amoxicillin, *CTRX* ceftriaxone, *ST* sulfamethoxazole-trimethoprim, *MINO* minocycline, *LVFX* levofloxacin, *MTNZ* metronidazole, *IPM/CS* imipenem/cilastatin, *FOM* fosfomycin, *CPFX* ciprofloxacin, *ND* no data

Resection of the infected aorta and in situ graft replacement, together with long term intravenous antibiotic therapy is considered to be standard care. The mortality rate associated with surgical treatment ranges from 11 to 36% [15]. In 1998, Semba et al. [16] reported successful treatment with endovascular aneurysm repair in mycotic aneurysm for the first time. Razavi et al. [15] reported that the short term outcome associated with the use of stent-grafts appeared to be better than that associated with graft replacement, although late aneurysm-related events were still frequent. A systematic review of outcomes after endovascular stent-grafts for mycotic aneurysm shows that the 30-day survival rate is 89.6 ± 4.4%, and the 2-year survival rate is 82.2 ± 5.8% respectively [17]. Although standard therapy still consists of resection of the infected aorta and in situ graft replacement, endovascular stent grafting might be an alternative therapy in patients who are not candidates for surgical replacement due to underlying diseases, as our case suggests.

Despite the given antibiotic susceptibility pattern, our patient’s bacteremia persisted for more than a week. A previous report shows that ruptured aneurysms or fever at time of stent-graft placement are significant predictors for persistent infection [17]. Lack of elimination of infection foci explains the persistence, but her underlying disease such as cirrhosis might have contributed too. Janda et al. reported that liver diseases such as alcoholic cirrhosis, ethanol abuse, hepatoma, hepatosplenomegaly, and icterus were frequently found in patients with *E. tarda* bacteremia [1]. Bacteremia alone is a cause of high mortality, and the presence of cirrhosis is an independent risk factor resulting in even worse mortality [18]. Underlying liver disease has not been shown to be associated with persistent bacteremia, but we need further study to verify this possibility.

*E. tarda* is susceptible to many antibiotics with minor exceptions. β-lactam antibiotics such as cephalosporins, aminoglycosides, and fluoroquinolones are usually effective [19], although it is resistant to colistin and polymyxin B [20]. Interestingly, although *E. tarda* produces beta- lactamase, a beta lactam resistant strain has not been reported so far [19]. The mortality of *E. tarda* bacteremia is high despite appropriate antibiotic therapy, and it makes optimal antibiotic choice difficult [19]. There are in vitro data suggesting that combination therapy of β-lactam and gentamicin might be more effective than monotherapy [21]. We treated our patient with combination therapy of ampicillin and gentamicin because of persistent fever and bacteremia with ampicillin monotherapy. Two days after starting combination therapy, blood cultures became sterile. The present case developed bacteremia again only after 1 week following discontinuation of antibiotics. Therefore, we planned to continue amoxcillin/clavulanate indefinitely.

This is a first case report of mycotic aneurysm caused by *E. tarda* successfully treated with stent graft insertion and prolonged antibiotic therapy without resection and debridement. Further investigations are needed to verify our approach is effective in other patients. Further studies also will be needed to investigate the value of combination antibiotic therapy, along with the evaluation of optimal duration of antibiotics.

## Additional file

**Additional file 1.** Database strategies.

## Abbreviation

TEVAR: thoracic endovascular aortic repair
