# Supplementary material for: Mycotic aneurysm caused by Edwardsiella tarda successfully treated with stenting and suppressive antibiotic therapy: a case report and systematic review
Source: Ann Clin Microbiol Antimicrob. 2018 May 10;17:21. doi: 10.1186/s12941-018-0273-x (PMC5944098; doi:10.1186/s12941-018-0273-x)
Supplement: Supplementary file 1 — Additional file 1. Database strategies. [file 12941_2018_273_MOESM1_ESM.docx]

Supplementary Appendix.: Database strategies

**PubMed**

#1 Edwardsiella tarda

#2 aneurysm

#3 vascular prosthesis

#4 stent graft

#5 #2 or #3 or #4

#6 #1 AND #5

2018/3/14, 2 articles

**Google Scholar**

1. "Edwardsiella tarda" AND ("aneurysm" OR "vascular prosthesis" OR "stent graft")

2018/3/14, 88 articles

2. "Edwardsiella tarda" AND ("動脈瘤" OR "人工血管" OR "ステントグラフト")

(We used Japanese characters in the search.)

2018/3/14, 8 articles

**Ichushi-Web**

((Edwardsiella/TH or Edwardsiella/AL) and tarda/AL) and (((動脈瘤/TH or 動脈瘤/AL)) or ((人工血管/TH or 人工血管/AL)) or ((ステント/TH or ステントグラフト/AL)))

(We used Japanese characters in the search.)

2018/3/14, 3 articles
